# Predictive Biomarkers of Response to Immunotherapy in Metastatic Renal Cell Cancer

**DOI:** 10.3389/fonc.2020.01644

**Published:** 2020-08-12

**Authors:** Alessandra Raimondi, Pierangela Sepe, Emma Zattarin, Alessia Mennitto, Marco Stellato, Melanie Claps, Valentina Guadalupi, Elena Verzoni, Filippo de Braud, Giuseppe Procopio

**Affiliations:** ^1^Department of Medical Oncology, Fondazione IRCCS Istituto Nazionale dei Tumori, Milan, Italy; ^2^Oncology and Hemato-Oncology Department, University of Milan, Milan, Italy

**Keywords:** biomarker, immunotherapy, metastases, PD-L1, renal cell carcinoma, tumor mutational burden

## Abstract

**Introduction:**

In the last decades, the therapeutic decision-making approach to metastatic renal cell cancer (mRCC) has dramatically changed thanks to the introduction in the treatment scenario of, first, anti-angiogenic agents and, afterward, immune-checkpoint inhibitors (ICIs). Immunotherapy is now the standard of care in pretreated mRCC patients and has recently entered even the first line setting. Nevertheless, in mRCC as well as in other tumor settings, a durable and clinically meaningful benefit from treatment with ICIs is not obtained for all patients treated. Therefore, the necessity to identify and validate predictive biomarkers of response to immunotherapy has emerged, in order to design the optimal treatment strategy for mRCC patients.

**Discussion:**

In this review, we present and discuss the most promising predictive biomarkers of response to ICIs in mRCC with the recent data available. In details, the first marker that was investigated is the immunohistochemical expression of programmed death receptor ligand 1 (PD-L1), showing a negative prognostic role in mRCC, but the debate about its potential predictive value is still open. Additionally, the high heterogeneity in PD-L1 determination methods adds complexity to this issue. Second, the tumor mutational or neoantigen burden is an emerging biomarker of increased response to immunotherapy, hypothesizing that the higher the TMB, the higher is the production of neoantigens, and thus the stimulation of anti-tumor immune response, even though controversial results have been obtained. Third, the tumor microenvironment, namely the different populations of the immune infiltrate, plays a key role in tumor progression and in the response to immunotherapy. Finally, several studies have collected evidence on the potential association of the occurrence of immune-related adverse events (irAEs) with the benefit from ICIs, first in non-small cell lung cancer (NSCLC) and melanoma, and recently even in mRCC.

**Conclusion:**

Several promising biomarkers of response to immunotherapy with ICIs have been identified, though without conclusive results upon their potential predictive value in mRCC. Therefore, the results of the exploratory analyses of the recently presented first-line trials and hopefully of future prospective, biomarker-driven studies could provide useful tools to be applied in the everyday clinical practice.

## Introduction

Renal cell cancer (RCC) represents about 3% of all solid tumors worldwide, with an estimated incidence of approximately 330,000 new cases per year (1). RCC is diagnosed at an advanced stage in around one third of cases, and metastases develop in another 30% of patients after initial nephrectomy for localized disease (2). The prognosis of metastatic RCC (mRCC) is generally considered poor, with a predicted 5-year survival rate lower than 20%, even though the patients’ outcome could be stratified according to several prognostic factors (3).

In the past decades, thanks to a deeper understanding of the biological and molecular disease scenario, the clinical approach to mRCC has undergone a dramatic change with the introduction of novel drugs (4). First, the anti-angiogenic therapies targeting the Vascular Endothelial Growth Factor (VEGF) and its receptor, and, subsequently, immunotherapy revolutionized the armamentarium for the treatment of mRCC. Therefore, the refined therapeutic decision-making process allowed to reach a remarkable improvement in patients’ outcome in terms of progression-free survival (PFS) and overall survival (OS), as well as in patients’ quality of life (5, 6).

In details, the targeting of the immune system deeply changed the disease course and the prognosis of patients affected by several tumors, among which non-small cell lung cancer (NSCLC), melanoma and RCC. The introduction of the immune-checkpoint inhibitors (ICIs) targeting first the Cytotoxic T-Lymphocyte Antigen 4 (CTLA4) and subsequently the programmed death receptor 1 (PD1) and its ligand (PD-L1) provided a new therapeutic standard and a changing paradigm in different tumor settings (7).

For what concerns mRCC, immunotherapy entered the clinical scenario in patients who had received a prior line with anti-angiogenic agents, in light of the results of the CheckMate 025 trial (8, 9). The study compared the anti-PD1 agent nivolumab with the standard of care everolimus and showed a statistically significant and clinically meaningful improvement in terms of survival with a more favorable safety profile for immunotherapy, leading to the approval from Food and Drug Administration (FDA) and European Medical Association (EMA) (10, 11).

Subsequently, in order to optimize the potential benefit of immunotherapy, ICIs have been investigated in the first-line setting, either alone (i.e., nivolumab plus ipilimumab) or associated with anti-vascular agents (i.e., avelumab plus axitinib, pembrolizumab plus axitinib and atezolizumab plus bevacizumab), thanks to several clinical trials that recently presented their first results (12). Altogether, the available data showed an overall improvement of the treatment outcomes with immunotherapy-based combinations over the anti-vascular tyrosine kinase inhibitor (TKI) monotherapy. In details, in the CheckMate 214 trial, a significant benefit in OS and overall response rate (ORR) for nivolumab plus the anti-CTLA4 agent ipilimumab over sunitinib was reported for intermediate/poor risk patients (13), and the Javelin Renal 101 study showed a 6.6-month increase in PFS for the anti-PD-L1 agent avelumab combined with axitinib as compared to sunitinib monotherapy (14). Moreover, in the IMmotion151 trial, the combination of the anti-PD-L1 atezolizumab plus bevacizumab prolonged PFS as compared to sunitinib in patients with PD-L1 positive mRCC (15), and the KEYNOTE-426 study demonstrated a significant benefit in terms of OS, PFS and ORR for the anti-PD1 agent pembrolizumab plus axitinib versus sunitinib (16). In light of these results, the treatment paradigm for mRCC patients is undergoing a deep change, in particular in the first-line setting, since FDA and EMA recently approved the combination of nivolumab plus ipilimumab for intermediate and poor risk patients and the association of pembrolizumab plus axitinib independently from the risk category (17, 18).

Additionally, studies are underway to investigate whether a rechallenge with ICIs in later lines could be safe and effective. Initial evidence has been collected on this topic, suggesting that this strategy might be feasible, even though prospective *ad hoc* studies should be designed and conducted in order to potentially validate this therapeutic approach in mRCC patients (19).

Nevertheless, the benefit of immunotherapy is confined to a limited proportion of mRCC patients who achieve a significant and durable disease control from treatment with ICIs. Therefore, the identification of predictive biomarkers for response to immunotherapeutic agents represents an unmet clinical need in this disease setting, with the aim to improve the outcome of mRCC patients.

**FIGURE 1:**
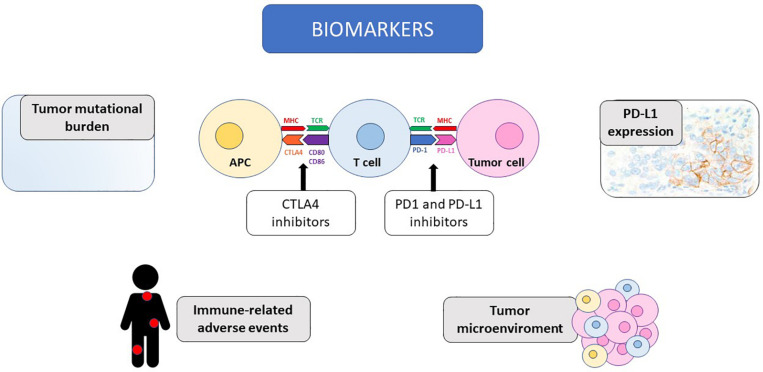
The potential biomarkers of response to immunotherapy discussed in the review are illustrated, together with the mechanism of action of immune-checkpoint inhibitors. CTLA4, Cytotoxic T-Lymphocyte Antigen 4; PD-1, programmed death receptor 1; PD-L1, programmed death receptor ligand 1; MHC, major histocompatibility complex; TCR, T cell receptor; APC, antigen presenting cell; CD, cluster of differentiation.

In this review, we summarize the most promising predictive biomarkers for response to immunotherapy and the available evidence collected on this topic in the setting of mRCC, as illustrated in Figure 1.

## Biomarkers

### PD-L1

The first and most studied potential biomarker for response to anti-PD1/PD-L1 agents is the expression of PD-L1. The clinical significance of the expression of PD-L1 on tumor cells and/or on the immune cells infiltrating the tumor assessed with immunohistochemistry (IHC) was initially identified in the first clinical study investigating the anti-PD1 agent nivolumab and, afterward, it has been widely studied in several settings (20).

The reported rate of PD-L1 expression in the different tumors is endowed with a high variability across studies, and, considering specifically the neoplastic diseases with enhanced response to ICIs, namely NSCLC, melanoma and RCC, it ranges between 14 and 100% (21). Nevertheless, even neoplasms with reduced sensitivity to immunotherapy, for example colorectal cancer or sarcoma, show a comparable IHC expression of PD-L1, thus underlining the limitations of this biomarker and giving an insight on the complexity of the scenario (22).

Considering the association of the expression of PD-L1 with prognosis, several studies collected evidence on a potential negative prognostic value of PD-L1 overexpression in gastric, hepatocellular, esophageal, pancreatic, ovarian, and bladder cancer, whereas a positive value in breast cancer and merkel cell carcinoma and controversial results in lung cancer, colorectal cancer, and melanoma (23). For example, in advanced/metastatic NSCLC, a systematic literature review did not observe an association between PD-L1 expression and patients or tumor characteristics but, in the majority of studies, a high expression of PD-L1 correlated with reduced survival (24). Nevertheless, the global level of evidence does not allow to drive definitive conclusions on this topic.

In the setting of mRCC, the negative prognostic role of the expression of PD-L1 on tumor cells has been reported in several studies (25, 26). Renal cancers showing the expression of PD-L1 on tumor cells display a higher tumor stage, a worse response to TKI therapy and a poorer prognosis (27). In details, in two *post hoc* analyses of the COMPARZ study and the METEOR and CABOSUN trials, respectively, PD-L1 expression on tumor cells was associated with shorter survival in mRCC patients, irrespectively of the type of targeted therapy received (28, 29). Furthermore, in a metanalysis including more than 1300 patients, the expression of PD-L1 in RCC seemed to confer a poorer outcome, approximately doubling the risk of death, and this finding was confirmed after the restriction of the analysis to patients affected by clear cell histology RCC, in case of advanced disease and in cases in which PD-L1 was evaluated uniquely with IHC (30).

On the other hand, dealing with the predictive value of PD-L1 expression for an enhanced response to immunotherapy with PD1/PD-L1 inhibitors, the global burden of evidence collected in several tumors showed that a higher expression of PD-L1 may be able to predict the responsiveness to ICIs, even though in most cancers the relevance of this correlation is insufficient to validate PD-L1 as a predictive biomarker potentially applicable in the clinical setting (31, 32). In melanoma, in trials investigating nivolumab and pembrolizumab in the advanced/metastatic setting, patients whose tumors displayed the positivity for PD-L1 had a higher response rate and a prolonged survival outcome, however, robust and durable responses to treatment were observed even in a considerable proportion of PD-L1 negative patients (33, 34). Therefore, PD-L1 expression is not currently used in the clinical practice as a biomarker to guide patients’ selection for treatment. On the other hand, in advanced NSCLC, the results of the recent studies evidenced how the IHC expression of PD-L1 and its cutoff are fundamental in the therapeutic decision-making of the non-oncogene-addicted disease, whether choosing the anti-PD1 monotherapy, chemotherapy or the combination of chemotherapy and immunotherapy in first-line (35).

In mRCC, the potential predictive value for response to immunotherapy of PD-L1 IHC expression is still controversial and the results obtained in the exploratory analyses of the landmark clinical trials investigating ICIs in this disease are inconclusive (8, 13–16). In details, in the CheckMate 025 trial, that proved the superiority of nivolumab over the standard of care everolimus in pre-treated patients, the expression of PD-L1 was associated with poor survival independently from the treatment received, but not with response to nivolumab. In details, in patients with PD-L1 ≥ 1% assessed on tumor cells, an inferior OS was observed as compared to those with PD-L1 < 1% in both treatment arms, with a similar trend for the 5% cutoff. On the other hand, nivolumab was superior to everolimus irrespectively of PD-L1 expression, since median OS resulted to be 21.8 versus 18.8 months and 27.4 versus 21.2 months in PD-L1 ≥1 and <1% subgroups, respectively (8). Moving to the first-line setting, in the CheckMate 214 trial, a longer PFS was observed for the combination immunotherapy ipilimumab plus nivolumab over sunitinib in IMDC intermediate-poor risk patients for PD-L1 positive tumors, that is 1% or greater (median PFS 22.8 versus 5.9 months), but not for negative ones, that is less than 1% (median PFS 11 versus 10.4 months). Conversely, an improvement in ORR and OS for immunotherapy over the anti-vascular agent was reported independently from the tumor PD-L1 expression level, even though the magnitude of benefit was higher in patients with PD-L1 ≥1% (13). Recently the phase 3 IMmotion151 trial reported that the combination of atezolizumab plus bevacizumab was superior to sunitinib monotherapy in terms of PFS in patients affected by PD-L1 positive mRCC, with a more favorable safety profile. The trial included patients independently from the PD-L1 status, that represented a stratification factor, and the magnitude of benefit seemed increased in patients with PD-L1 ≥1% as compared to the intention-to-treat trial population (15). These evidences are in line with the results of the phase 2 IMmotion150 study, that was designed with two atezolizumab-based arms (monotherapy or in combination with bevacizumab) as compared to sunitinib, and evidenced a trend in a progressively increasing treatment efficacy with higher PD-L1 expression (defined as any intensity staining in immune cells covering absent/<1% (IC0), ≥1% to <5% (IC1), ≥5% to <10% (IC2) or ≥10% (IC3) of tumor area assessed by SP142 assay) mainly in the atezolizumab/bevacizumab arm (36). Furthermore, in the JAVELIN Renal 101 trial, where PD-L1 positivity was assessed on immune cells infiltrating the tumor with a cutoff of 1%, the combination of avelumab plus axitinib showed a PFS and ORR advantage as compared to sunitinib monotherapy, and the benefit was substantially comparable in the overall population and in the PD-L1 positive subgroup (median PFS 13.8 versus 8.4 months and 13.8 versus 7.2 months; ORR 51.4% versus 25.7% and 55.2% versus 25.5%, in avelumab plus axitinib versus sunitinib arms, respectively), even though with a non-significant trend toward improved PFS in the PD-L1 negative group (14, 37). Finally, in the KEYNOTE-426 trial, the combination of pembrolizumab plus axitinib showed a benefit over sunitinib in terms of PFS, OS and ORR, irrespectively of PD-L1 expression (assessed with the combined score, including tumor cells, lymphocytes and macrophages), although the hazard ratios for PFS and OS seemed to underline an enhanced advantage in PD-L1 ≥1 versus <1 subgroups [0.62 (95%CI 0.47–0.80) versus 0.87 (95%CI 0.62–1.23) for PFS and 0.54 (95%CI 0.35–0.84) versus 0.59 (95%CI 0.34–1.03) for OS] (16).

Overall, these results suggest that the expression of PD-L1 in mRCC is not able to completely predict the potential responsiveness of tumor to anti PD1/PD-L1 ICIs, thus, the role of PD-L1 as a predictive therapeutic biomarker remains controversial and warrants further investigation in *ad hoc* designed studies.

The main limitations of PD-L1 expression are related both to the assessment method and to the tumor heterogeneity (22). The first weakness, connected with the technique used for the IHC analysis, is the elevated variability among the different determination methods available, since each PD1/PD-L1 inhibitor is endowed with its own companion antibody (e.g., Dako, Leica platform, Ventana Medical System). In details, the scoring systems are not homogeneous for the target cells assessed, whether tumor cells, immune cells infiltrating the neoplastic stroma or the combination of both, and the positivity cutoff has not been validated (e.g., 1, 5, and 10%) (32, 38). Additionally, it is not clear whether the expression of PD-L1 or PD1 should be assessed, and, additionally, the role of PD-L2, the other ligand of PD1, is still to be fully elucidated (39). Finally, the expression of PD-L1 should not be considered static but dynamic, since it may vary during the tumor natural history or as a consequence of anti-neoplastic treatments and, additionally, PD-L1 expression shows an elevated heterogeneity both intratumoral and between the primary tumor and the distant metastases, potentially explaining the high responses obtained in cases in which the primary tumor or one lesion had been characterized as PD-L1 negative (40).

### Tumor Mutational Burden/Neoantigen Burden

The tumor mutational burden (TMB) is a recently defined emerging biomarker of increased response to immunotherapy. The definition of TMB is the total number of mutations per coding area of tumor genome, measured as mutations per megabase (mutations/Mb). The genomic alterations occurring in tumor cells display the capacity to generate tumor-specific antigens (neoantigens), potentially processed and presented on the surface of cancer cells and therefore recognized by the T cells, eliciting the anti-tumor response (41). The potential association of TMB with sensitivity to ICIs is based on the hypothesis that in tumors with high TMB there is an increased production of surface neoantigens, thus stimulating the anti-tumor immune system response (42). TMB has been investigated in several tumor settings, mainly NSCLC and melanoma, as a stratification marker to predict the response to immune agents, showing promising yet inconclusive results (43, 44). In details, in the CheckMate 227 trial, patients with NSCLC were randomized to receive different nivolumab-based regimens versus chemotherapy and the results showed that those displaying a TMB equal or higher than 10 mutations/Mb had an increased PFS with the treatment combining nivolumab and ipilimumab (43). The non-conclusive results obtained may be partially explained by the heterogeneity in the definition and assessment methods of TMB, as well as of positivity cutoffs, since TMB could be assessed by the estimation of somatic mutations with whole exome sequencing or by the direct sequencing of panels of cancer-related genes using next-generation sequencing. Moreover, these techniques are not widely available, limiting the potential application of this tool in the clinical practice (45).

In the setting of mRCC, initial evidence has been collected on this topic in recent studies. TMB scores display a deep variability through the different histological subtypes of RCC, ranging from a very low level in chromophobe type to a comparable value of TMB in clear cell and papillary tumors and, additionally, TMB is not associated with the clinically defined prognostic groups according to IMDC and MSKCC (46). Interestingly, despite its high rate of response to immunotherapy, RCC is endowed with an average low TMB, only 1.1 mutations/Mb, as compared with melanoma and NSCLC that display values ranging from 10 to 400 mutations/Mb (46, 47). Nevertheless, RCC represents the tumor endowed with the highest proportion of insertion/deletion alterations, that represent a highly immunogenic mutational class, thus potentially leading to the production of neo-antigens (48). The results regarding the prognostic value of TMB and its potential correlation with response to immunotherapy in the setting of mRCC are conflicting. Regarding the association with prognosis, the literature evidences are controversial, since some studies report a trend toward improved survival with the increase of TMB, while others observed a negative prognostic value (49, 50). Concerning the predictive value of TMB, TMB and other biomarkers were evaluated in a subset of 35 pre-treated mRCC patients enrolled in the phase 1b study of nivolumab (NCT01358721), showing that only a specific and small proportion of tumors could benefit from treatment, and the results were then validated in another independent cohort of patients treated with nivolumab or atezolizumab (51). Moreover, in a retrospective dataset of 34 mRCC patients, TMB did not correlate neither with survival nor with PD-L1 expression on tumor cells (52). Additionally, in the exploratory analysis of the IMmotion150 trial, no association between TMB or neoantigen burden with an enhanced clinical benefit from nivolumab was shown (36).

Taken all these data into consideration, *ad hoc* prospective trials are awaited, with the aim to unravel the potential predictive role of TMB in the setting of mRCC. The ongoing NIVES study (NCT03469713), investigating the combination of nivolumab with stereotactic radiotherapy in pretreated mRCC patients, recently presented the first data, and its exploratory analyses could provide evidence on this topic in the next future (53).

### Microenvironment/Signatures

In the complex scenario of cancer evolution and of response to immunotherapy, the tumor microenvironment plays a key role, since the different subpopulations of immune cells that infiltrate the tumor and their interplay may deeply influence the sensitivity to ICIs.

Renal cell cancer is endowed with a heterogeneous population of tumor infiltrating immune cells, that include T and B lymphocytes as well as innate immunity cells like monocytes and macrophages. A remarkable burden of evidence has been collected on the characterization of the tumor infiltrate in RCC, highlighting its impact on patients’ clinical outcome and suggesting a potential role as a target in cancer treatment (54). However, conflicting data have been obtained as far as now. In details, some studies have shown that the infiltration of effector T cells, such as CD8+ lymphocytes, and M1 macrophages may confer a better prognosis, whereas regulator T cells, such as T regs, and M2 macrophages could be associated with a poorer outcome (55–58). Conversely, other trials have reported a poor prognosis in cases displaying a high intra- and peri-tumoral T CD8+ cells density, potentially related to the positive association between lymphocytes and tumor grade (59). Interestingly, it was reported that the expression of PD-L1 on tumor cells correlated with a higher infiltration of T CD8+ cells in the microenvironment of RCC, with potential implications for prognosis (54), that is influenced even by the presence and grade of activation of dendritic cells in the tumor, able to drive the anti-tumor immune reaction (60).

Concerning the potential predictive role of the immune infiltrate, in several tumors (such as melanoma, breast and colorectal cancer), it was reported that the presence of tumor-infiltrating lymphocytes is associated with an enhanced response to immunotherapy (61). Moreover, moving forward, specific genomic signatures, based on gene expression profiles predictive of immune system activation and anti-tumor response, have been developed and investigated in various cancer settings as potential biomarkers able to predict the response to immunotherapy (62). In patients with solid tumors treated with pembrolizumab in a clinical trial setting, higher T cell–inflamed gene expression profile scores were associated with increased response to treatment (63). In mRCC, a comprehensive analysis on patients enrolled in four clinical trials with nivolumab showed that the expression profiles involved in the regulation of metabolic and immune pathways correlated with the clinical outcome. In details, on the one hand, the overexpression of genes involved in metabolic functions (e.g., UGT1A) and, on the other, the increased expression of immune markers (e.g., BACH2 and CCL3) correlated with a poorer and higher response to treatment, respectively (64). Additionally, the exploratory analysis of the IMmotion150 trial reported that a T-effector immune gene signature displayed an association with the expression of PD-L1 and the tumor infiltration of T CD8 + cells, resulting in an increased response to atezolizumab, with a higher ORR and a prolonged PFS (36). These data find their rationale in the potential immune-modulating reaction induced by the vascular inhibition via VEGF-blockade, able to promote the infiltration of T cells in the tumor microenvironment, thus potentiating the mechanism of action of ICIs (65). Further studies on this topic are awaited, given the complexity of the molecular and biological cancer scenario in mRCC.

### Immune-Related Adverse Events

Another emerging topic in the setting of the identification of potential predictors of response to immunotherapy is the occurrence of immune-related adverse events (irAEs). In details, ICIs determine a peculiar spectrum of treatment-related toxicities, arising from an excessively augmented activity of the immune system and potentially involving any organ with a multifaceted variety of presentations (66, 67). The pathogenesis of irAEs may arise from the cross-reactivity between normal tissues and tumor neoantigens or from the alteration of the humoral and/or cell-mediated immunity, including immunosuppressive regulatory T cells and the production of antibodies by B cells. The most common ICIs-related toxicities include dermatological AEs, such as rash, vitiligo and pruritus, gastrointestinal, like diarrhea, colitis, hepatitis, amylase and lipase elevation, endocrine, such as thyroid disfunctions (hypothyroidism and/or hyperthyroidism) and hypophysitis, pulmonary, as pneumonitis, renal and systemic, like fever and fatigue (68). For what concerns specifically the renal function impairment, the main irAE is nephritis, either symptomatic or not, that consists in an inflammation of the kidney affecting the structure and limiting its functions. This is peculiarly relevant for RCC patients, since they may have a baseline reduction of renal function or a limited functional reserve. The guidelines for the management of immune-related nephritis are based upon the grading of the AEs and recommend to stop or even discontinue the treatment with ICIs, consult a nephrology specialist and, in selected cases, to start a corticosteroid treatment (67). A remarkable yet non-conclusive amount of data has been collected upon a possible correlation between the occurrence of irAEs and an increased benefit in terms of survival from ICIs, mainly in melanoma and NSCLC (69–71). Recently, even in the setting of mRCC, some studies have provided evidence of a consistent result. In details, in mRCC patients treated with nivolumab in the second and further line setting in a compassionate-use program, those reporting irAEs showed a significantly longer OS as compared to patients not experiencing immune-mediated toxicity (median OS not reached versus 16.8 months) (72). Comparable results have been obtained in a retrospective dataset of Asian patients with mRCC receiving nivolumab, highlighting how irAEs were associated with improved PFS and OS (73).

Nevertheless, the specific criteria for the definition of irAEs have not been established so far and whether any toxicity arising from an immune-mediated mechanism or specific classes of irAEs display a meaningful correlation with response to immunotherapy remains to be elucidated yet, thus warranting further investigation in this setting.

## Conclusion

Immunotherapy revolutionized the therapeutic strategy and the clinical outcome of patients affected by several different tumors, among which RCC. Nevertheless, not all patients derive a significant and durable benefit from immunotherapy with ICI, indeed, this underlines the unmet clinical need of identifying reliable predictive markers of response to immune agents. The development of biomarkers endowed with high sensitivity, specificity and accuracy able to identify which patients may truly benefit from the treatment with ICIs would allow to refine the therapeutic selection and to better tailor the treatment strategy. Furthermore, recent studies provided evidence that maybe even the prognostic stratification of mRCC patients should be refined in those treated with ICI, including the role of systemic inflammation besides the canonical criteria (74).

The amount of data collected so far in the setting of mRCC, encompassing the expression of PD-L1, the TMB and the widespread role of immunity, has not yet established a predictive biomarker applicable in the clinical practice. The first remark is that probably the huge complexity of the scenario of response to immunotherapy could not be captured by a single biomarker, thus potentially suggesting the need for more comprehensive and integrated approaches. Another point to underline is that the high heterogeneity of the studies conducted, as well as of the techniques and methodologies used and the variety of markers investigated, adds further complexity and limits the validation of these biomarkers with the aim to realize a widely exploitable tool in the clinical setting. Finally, although extremely promising results have been obtained for several biomarkers so far, solid evidence-based data deriving from large, prospective *ad hoc* clinical trials should be collected in order to change the clinical decision-making process in the everyday real-life.

## Author Contributions

AR and GP conceptualized and designed the manuscript. AR drafted the manuscript. PS, EZ, AM, MS, MC, VG, EV, FB, and GP critically reviewed the manuscript. All the authors approved the final version of the manuscript submitted.

## Conflict of Interest

GP declares receiving honoraria for advisory board from Bayer, Bristol Myers Squibb, Ipsen, Merck, Novartis, and Pfizer. EV declares receiving honoraria for advisory board from Pfizer, Bristol Myers Squibb, Ipsen, and EUSA pharm. FB reported receiving honoraria for speaker activities and participation in advisory boards from Amgen, Inc., Roche, and Novartis International AG. The remaining authors declare that the research was conducted in the absence of any commercial or financial relationships that could be construed as a potential conflict of interest.
